# Empty Follicle Syndrome Due to Failure of Gonadotropin-Releasing Hormone Agonist Trigger: A Retrospective Study of Possible Factors That Affect the Outcome of a Rescue Human Chorionic Gonadotropin Protocol

**DOI:** 10.7759/cureus.114086

**Published:** 2026-08-06

**Authors:** Daichi Inoue

**Affiliations:** 1 Department of Reproductive Medicine, Asada Ladies Clinic, Nagoya, JPN

**Keywords:** empty follicle syndrome, gnrh agonist (gnrh-a) trigger, gnrh antagonist protocol, hcg trigger, in vitro fertilization ivf, rescue hcg protocol

## Abstract

Introduction

Empty follicle syndrome (EFS) may occur in in vitro fertilization (IVF) cycles that use a gonadotropin-releasing hormone (GnRH) antagonist protocol with a single GnRH agonist trigger. We evaluated whether a rescue human chorionic gonadotropin (hCG) trigger can salvage oocyte retrieval in these cases, and identified factors associated with obtaining a transferable embryo.

Methods

Among 4,778 cycles triggered with a GnRH agonist alone between June 2014 and February 2019, we retrospectively identified 43 cycles, in 43 patients, in which no mature oocyte was obtained at the initial retrieval, and a rescue hCG protocol with repeat ovum pick-up was performed. Cycles were divided into an embryo-transfer (ET)-positive group, in which a transferable embryo was obtained (n = 30), and an ET-negative group (n = 13). Baseline and peri-trigger hormone levels were compared, and thresholds for baseline luteinizing hormone (LH) and follicle-stimulating hormone (FSH) were derived by receiver operating characteristic analysis and evaluated in contingency tables.

Results

The incidence of GnRH agonist trigger failure was 0.9% (43/4,778 cycles). Oocytes were collected in 37 of 43 cycles, and a transferable embryo was obtained in 30 (69.8%); frozen-thawed transfer resulted in 18 live births, a live birth rate of 41.9% per rescue cycle. A baseline LH threshold of 1.9 mIU/mL (area under the curve (AUC) 0.83) and a baseline FSH threshold of 5.4 mIU/mL (AUC 0.85) distinguished the two groups (P < 0.001 for both); the corresponding odds of failing to obtain a transferable embryo were 33.0 (95% CI 3.7-296) and 60.0 (95% CI 6.3-572), with confidence intervals reflecting the small number of events. All 43 patients developed ovarian hyperstimulation syndrome (OHSS): 28 mild and 15 moderate. No severe OHSS requiring hospitalization occurred.

Conclusions

A rescue hCG trigger is a workable management strategy for EFS following a GnRH agonist trigger, yielding live births in cycles that would otherwise have been cancelled. All patients in this series developed mild or moderate OHSS, and none developed severe OHSS, although with 43 cycles the series is too small to establish the risk of severe OHSS. Baseline LH and FSH, together with the change in estradiol and progesterone between the first and second triggers, may help estimate the probability of obtaining a transferable embryo, but these thresholds are exploratory and require prospective validation.

## Introduction

Ovarian hyperstimulation syndrome (OHSS) is one of the most important and potentially life-threatening complications of in vitro fertilization and embryo transfer (IVF-ET). Several strategies have been proposed to prevent it, including the use of a gonadotropin-releasing hormone (GnRH) agonist alone to trigger ovulation within a GnRH antagonist protocol [1,2], and coasting, in which gonadotropin stimulation is withheld while the trigger is deferred, although coasting for three days or more is reported to worsen outcomes [3]. The patients who benefit most from an agonist trigger include those with polycystic ovarian syndrome and elevated anti-Müllerian hormone (AMH) levels, in whom obstetric and neonatal complications may also be reduced [4]. In most cases, OHSS is avoided, and mature oocytes are safely retrieved [5], but occasionally no mature oocyte is obtained, a condition known as empty follicle syndrome (EFS).

Empty follicle syndrome is defined as the failure to retrieve oocytes after adequate ovarian stimulation and a technically correct attempt at ovarian puncture with follicular aspiration, and it is a recognized limitation of the GnRH agonist trigger [6,7]. It is conventionally classified into two types according to hormone levels on the day of oocyte retrieval: genuine EFS, in which retrieval fails despite a correctly administered trigger, whether human chorionic gonadotropin (hCG) or a GnRH analogue; and false EFS, in which retrieval fails because the trigger was not administered correctly [8]. The reported incidence ranges widely, from 0.045% to 7% [9], reflecting differences in definition as much as in practice.

Management of EFS is challenging for three reasons. First, the diagnosis is made only at the time of oocyte retrieval, when the patient has already completed a full course of stimulation and undergone follicular aspiration, so that the alternative to salvage is cancellation of a cycle in which the ovarian response has otherwise been adequate. Second, no consensus protocol exists: the condition is rare enough that published experience consists largely of case reports and small series [10-13]. Third, the salvage manoeuvre itself carries risk, since patients in whom a GnRH agonist trigger was selected are by definition those judged to be at high risk of OHSS, and rescue with hCG reintroduces precisely the exposure that the agonist trigger was chosen to avoid.

As a rescue alternative for EFS after a failed GnRH agonist trigger, we administer hCG intramuscularly as a second trigger on the day of the failed oocyte collection and repeat ovum pick-up (OPU) 34-36 h later. The dose is individualized within a range of 3,000-10,000 IU according to the estimated risk of OHSS, the lower doses being selected for patients with a high follicular count or a high serum estradiol, since a dose at the lower end of this range can achieve final oocyte maturation while limiting the luteotrophic exposure that drives OHSS [1,5]. This approach has enabled oocyte collection, embryo transfer, and live birth in selected patients [14,15].

Some patients develop EFS because of insufficient circulating hCG after an initial hCG trigger, and a second hCG trigger can rescue these cycles [16]. For EFS following a GnRH agonist trigger, Christopoulos et al. [10] reported a rescue hCG trigger in six patients, and Chang et al. [17] subsequently attempted re-triggering in 22 cycles. Although these findings support the usefulness of rescue hCG in selected cases, its overall effectiveness remains unclear, and factors predicting efficacy are needed to guide the selection of candidates.

We therefore retrospectively analyzed the performance of our rescue hCG protocol in patients in whom a GnRH agonist first trigger was deemed ineffective at the time of oocyte retrieval, and sought factors associated with the outcome.

## Materials and methods

Patients

**Figure 1:**
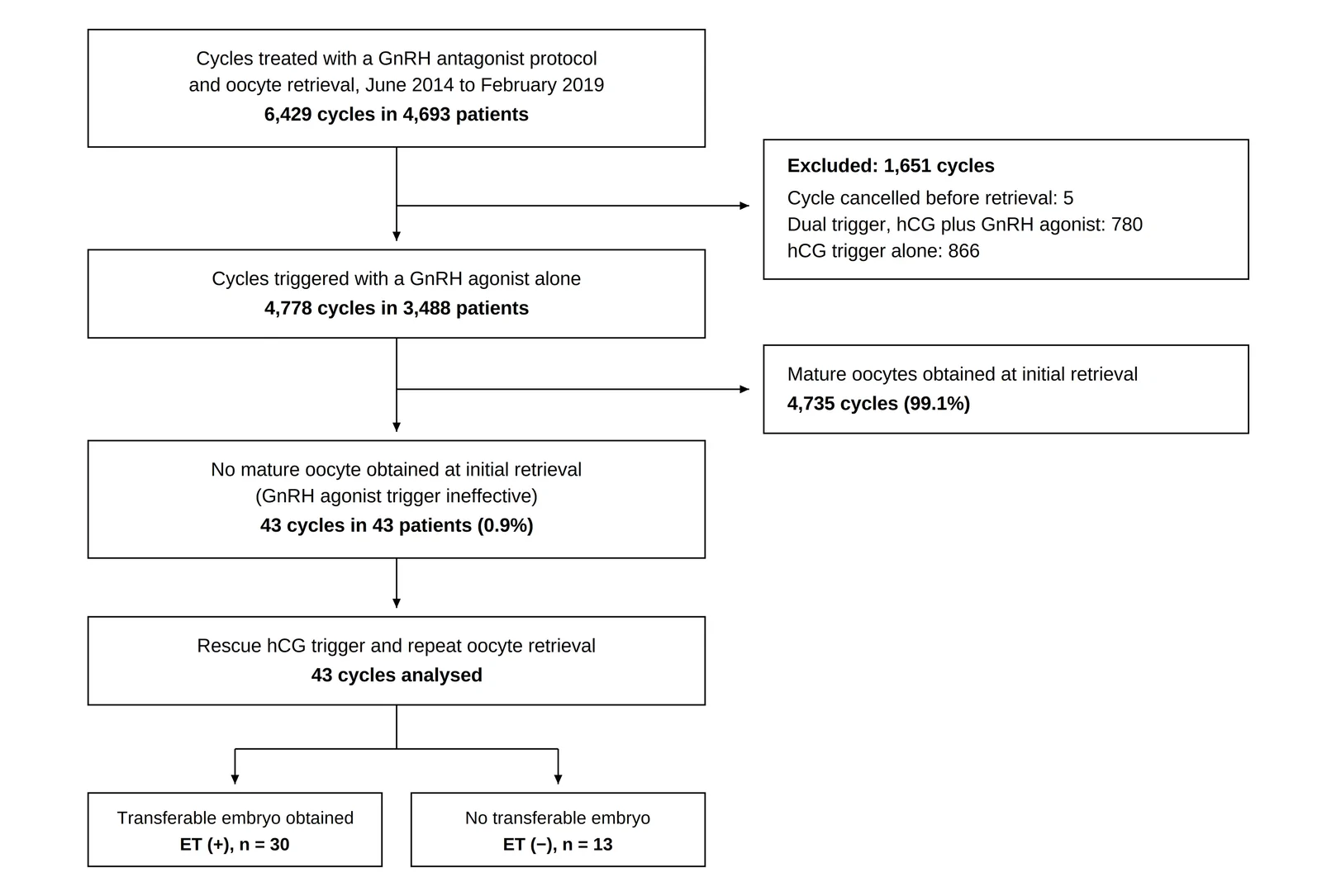
Selection of study cycles. Exclusions were applied at the cycle level. "Cancelled" denotes cycles in which oocyte retrieval was not attempted. Patient numbers are counts of distinct individuals and are not additive across the exclusion categories, since a patient could contribute cycles triggered by more than one method during the study period; patient totals are therefore given only for the whole cohort and for the analysed group. The 43 cycles with an ineffective GnRH agonist trigger were contributed by 43 different women, none of whom underwent repeat ovum pick-up more than once. GnRH, gonadotropin-releasing hormone; hCG, human chorionic gonadotropin; ET, embryo transfer.

Between June 2014 and February 2019, 6,429 cycles in 4,693 patients were treated with a gonadotropin-releasing hormone (GnRH) antagonist protocol and underwent oocyte retrieval at our hospital. Five cycles were cancelled before oocyte retrieval was attempted. Of the remaining cycles, 780 were triggered with a dual trigger of human chorionic gonadotropin (hCG) and a GnRH agonist, and 866 were triggered with hCG alone; both groups were excluded, since the present analysis concerns failure of a GnRH agonist trigger and neither is at risk of it. The study population therefore comprised the 4,778 cycles in 3,488 patients that were triggered with a GnRH agonist alone (Figure 1).

In 43 of these cycles (0.9%), no mature oocyte was obtained at the initial oocyte retrieval, and a rescue hCG protocol with repeat ovum pick-up (re-OPU) was performed. These 43 cycles constitute the study cohort. They were contributed by 43 different women; no patient underwent re-OPU on more than one occasion during the study period. Cycle and patient are therefore interchangeable in this analysis, and all observations are independent.

Exclusions were applied at the cycle level. The patient numbers reported above are counts of distinct individuals and are not additive across the excluded categories, since a patient could contribute cycles triggered by more than one method during the study period.

Collected data included age, body mass index (BMI), antral follicle count (AFC) of total bilateral ovarian follicles with a mean diameter of 2-10 mm on transvaginal ultrasound determined on cycle days (CDs) 2-5, and anti-Müllerian hormone (AMH) level obtained within 3 months of the start of controlled ovarian stimulation.

The study was approved by the institutional review board of Asada Ladies Clinic (approval 2019-09) and performed in accordance with the principles of the Declaration of Helsinki. Written informed consent was obtained from all patients prior to AMH measurement and disclosure of treatment results.

Controlled ovarian stimulation protocol

Ovarian stimulation was performed using recombinant follicle-stimulating hormone (FSH; Gonal-f, Merck Serono, Tokyo, Japan) 150-450 IU/body/day or human menopausal gonadotropin 150-450 IU/body/day (Ferring Pharmaceuticals, Tokyo, Japan; or ASKA Pharmaceutical, Tokyo, Japan) starting on CD 2-5. The GnRH antagonist ganirelix (Ganirest; MSD, Tokyo, Japan) or cetrorelix (0.25 mg; Cetrotide; Merck Serono) was administered when the dominant follicle reached a maximum diameter of approximately 14-16 mm, or when a premature luteinizing hormone (LH) surge was suspected. Final oocyte maturation was triggered with buserelin acetate (Buserecur; Fuji Pharma, Tokyo, Japan) 300 μg × 4 (transnasal) or leuprolide acetate (Lucrin; AbbVie, North Chicago, USA) 1 mg (subcutaneous) once the dominant follicle reached ≥18 mm and at least 13 days had elapsed since the start of stimulation. When OHSS risk was deemed low, the final trigger was hCG or a dual trigger of hCG and a GnRH agonist, selected on the basis of follicular diameter, serum estradiol (E2), patient age, and AMH; such cycles were excluded from the present analysis as described above.

Oocytes were collected 34-36 h after trigger under ultrasound-guided transvaginal aspiration. Retrieved oocytes were fertilized by conventional in vitro fertilization (IVF) or intracytoplasmic sperm injection (ICSI), with split insemination in cases with ≥6 metaphase II (M2) oocytes or oligozoospermia. All fertilized oocytes were cryopreserved at the pronuclear stage, and no embryo transfers were performed in the same cycle. When a sufficient number of fertilized oocytes were obtained, 4-12 were frozen at the pronuclear stage, taking the patient’s age into consideration, and the remainder were cultured to the blastocyst stage. In all cases, thawed embryo transfer was performed in a separate cycle using endometrial preparation with hormone replacement.

Rescue hCG (re-triggering) protocol

When the risk of ovarian hyperstimulation syndrome (OHSS) was judged to be high, oocyte retrieval was performed following a GnRH agonist trigger. Retrieval was initially attempted from one ovary only. If no mature oocyte was obtained from that ovary, retrieval was abandoned rather than extended to the contralateral ovary, and a second trigger of hCG was administered intramuscularly; retrieval from both ovaries was then repeated 34-36 h later (the rescue hCG protocol; re-trigger and re-OPU).

The decision not to aspirate the contralateral ovary at the initial procedure was a deliberate component of the protocol. Failure to obtain a mature oocyte from the first ovary was interpreted as a systemic failure of the trigger rather than a technical or side-specific failure, since an effective trigger acts on both ovaries simultaneously and a unilateral failure of maturation has no plausible mechanism. Aspirating the contralateral ovary at that point would have punctured follicles that remained capable of responding to a second trigger, thereby reducing the oocyte yield available at re-OPU, and would additionally have prolonged anaesthesia and increased the risk of haemorrhage from a second puncture of an enlarged ovary. The contralateral ovary was therefore left intact so that it could contribute to the rescue retrieval.

The dose of hCG used for the second trigger was not governed by a written protocol. It was selected by the treating physician from 3,000, 3,333, 5,000, and 10,000 IU on the basis of an overall clinical assessment of the individual patient’s risk of OHSS, taking into account age, BMI, AMH, the serum E2 concentration and follicle count, the serum progesterone (P4) concentration, and the number of follicles already aspirated at the abandoned first retrieval. The intention in every case was to administer the lowest dose judged sufficient to achieve final oocyte maturation. The 3,333 IU dose represents one-third of a 10,000 IU ampoule, which was divided in order to obtain a low dose without waste.

The doses administered were 3,000 IU in 24 cycles (55.8%), 3,333 IU in 10 (23.3%), 5,000 IU in five (11.6%), and 10,000 IU in four (9.3%); 34 cycles (79.1%) therefore received 3,333 IU or less. This distribution is reported as an observed characteristic of the series rather than as the output of a defined algorithm.

Fertilized oocytes from the rescue retrieval were again cryopreserved at the pronuclear stage, and a freeze-thawed embryo transfer was performed in a subsequent cycle.

Hormone assay

Serum E2 (pg/mL), FSH, LH, prolactin, P4 (ng/mL), and AMH were measured using a Cobas e 411 analyzer (Roche Diagnostics K.K., Tokyo, Japan) by automated electrochemiluminescence immunoassay.

On trigger days, blood was drawn at the patient’s clinic visit, 4-13 h before the scheduled time of trigger administration; the interval varied according to the time of the visit relative to the fixed trigger time. Two consequences follow for the interpretation of the LH values reported here. Serum LH on the day of the first trigger is a pre-trigger value that reflects pituitary status under antagonist suppression and carries no information about the LH response to the GnRH agonist. Serum LH on the day of the second trigger was drawn before hCG administration but after the GnRH agonist trigger, and therefore represents a late post-agonist value obtained well beyond the 8-12 h window in which the adequacy of an agonist trigger is conventionally assessed [18,19]. No blood was sampled within that window.

OHSS classification

A medical examination was performed 3-5 days after the second oocyte retrieval, and OHSS was graded according to the Royal College of Obstetricians and Gynaecologists Green-top Guideline No. 5 [20]. The third edition, published in February 2016, was the version in force throughout the study period and is the one applied here; a fourth edition was published in 2026, after the study was completed, and retains the same severity classification.

Data and statistical analysis

Cycles were divided into two groups: the ET (+) group, in which a transferable embryo was obtained, and the ET (−) group, in which no transferable embryo was obtained. The primary comparisons were specified as baseline characteristics (age, AMH, BMI, AFC); serum LH, FSH, E2, and P4 at the start of stimulation and on the days of the first and second triggers, together with the proportional change in E2 and P4 between the two triggers; the oocyte and embryo yield at re-OPU; and the incidence of OHSS by grade. Live birth was the principal clinical outcome.

Normality was assessed with the Shapiro-Wilk test. Normally distributed data are presented as mean ± standard deviation; non-normally distributed data are presented as median (range). Missing-data variables were excluded case-wise. Continuous variables were compared with the Mann-Whitney U test. Receiver operating characteristic (ROC) analysis of baseline LH and FSH identified thresholds for predicting the presence or absence of a transferable embryo, and the population was re-categorized according to these thresholds. Proportions were compared with Fisher’s exact test when the total n was <20, or 20-40 with at least one expected cell count <5, and with the chi-square test with Yates’ continuity correction when n ≥40 with all expected cell counts ≥5. Odds ratios for the dichotomized gonadotropin thresholds were calculated from the corresponding two-by-two tables, with 95% confidence intervals obtained by Woolf’s method. Analyses were performed with EZR (version 1.55), a modified version of R commander [21]; p < 0.05 was considered statistically significant.

No multivariable regression model was fitted. With 13 events, and with only one cycle below each gonadotropin threshold failing to yield a transferable embryo, the data exhibit quasi-complete separation, under which maximum-likelihood regression coefficients are unstable and their confidence intervals uninterpretable. The association is therefore reported directly from the contingency tables, without adjustment. The whole of this analysis is exploratory and hypothesis-generating rather than confirmatory: the thresholds were derived from the same data in which they are evaluated, so the reported areas under the ROC curve are apparent values that are optimistically biased; no internal validation was performed, and prospective validation in an independent cohort would be required before the thresholds could be used clinically.

## Results

Between June 2014 and February 2019, 4,778 cycles in 3,488 patients were triggered with a GnRH agonist alone within a GnRH antagonist protocol. In 43 of these cycles (0.9%), no mature oocyte was obtained at the initial retrieval, and the rescue hCG protocol was applied in all 43. The selection of cycles is shown in Figure 1.

Background characteristics of the 43 cycles were age 35.0 (27-42) years, AMH 3.90 (1.2-21.0) ng/mL, BMI 20.0 (15.8-27.8) kg/m², and AFC 7 (0-22), presented as median (range). The 43 cycles were categorized by outcome into an ET (+) group (n = 30) and an ET (−) group (n = 13). The two groups did not differ significantly in age, AMH, BMI, or AFC (Table 1).

**Table 1:** Background of cycles treated with the rescue human chorionic gonadotropin protocol. Data are presented as median (range). All p values were calculated using the Mann-Whitney U test; the corresponding U statistic is given in the penultimate column. Statistical significance was defined as p < 0.05. No comparison in this table reached statistical significance. ET, embryo transfer; AMH, anti-Müllerian hormone; BMI, body mass index.

	ET (+) (n = 30)	ET (−) (n = 13)	U value	p value
Age (y)	34.0 (27–42)	35.0 (29–40)	180.5	0.65
AMH (ng/mL)	4.2 (1.2–20.9)	3.4 (1.2–21.0)	146.0	0.18
BMI (kg/m²)	20.0 (15.8–27.8)	20.1 (17.5–25.0)	184.5	0.79
Antral follicle count (n)	7.5 (0–22)	5.0 (0–11)	176.0	0.63

Hormone levels

Baseline LH at the start of stimulation was 0.3 (0.1-8.5) mIU/mL in the ET (+) group and 5.0 (3.2-12.0) mIU/mL in the ET (−) group (P < 0.001), and baseline FSH was 1.2 (0.1-11.9) and 8.1 (0.1-16.1) mIU/mL, respectively (P < 0.001). On the day of the first trigger, LH differed between the groups (P = 0.002) while FSH, E2, and P4 did not. On the day of the second trigger, LH, E2, and P4 all differed significantly while FSH did not. Full values are given in Table 2.

**Table 2:** Hormone values by outcome. Data are presented as median (range). All p values were calculated using the Mann-Whitney U test; the corresponding U statistic is given in the penultimate column. Statistical significance was defined as p < 0.05, and significant values are marked with an asterisk (*). Samples on trigger days were drawn 4-13 h before the scheduled trigger; the value on the day of the first trigger therefore precedes administration of the GnRH agonist, and that on the day of the second trigger follows it. ET, embryo transfer; CD, cycle day; sLH, serum luteinizing hormone; sFSH, serum follicle-stimulating hormone; sE2, serum estradiol; sP4, serum progesterone; GnRH, gonadotropin-releasing hormone.

	ET (+) (n = 30)	ET (−) (n = 13)	U value	p value
sLH on CD 2–5 (mIU/mL)	0.3 (0.1–8.5)	5.0 (3.2–12.0)	48	<0.001*
sLH on day of first trigger (mIU/mL)	0.1 (0.1–1.1)	0.3 (0.1–2.9)	86	0.002*
sLH on day of second trigger (mIU/mL)	0.4 (0.1–33.2)	1.3 (0.1–4.3)	132	0.04*
sFSH on CD 2–5 (mIU/mL)	1.2 (0.1–11.9)	8.1 (0.1–16.1)	41	<0.001*
sFSH on day of first trigger (mIU/mL)	24.2 (14.1–40.5)	24.0 (11.4–41.7)	183	0.54
sFSH on day of second trigger (mIU/mL)	19.4 (7.3–31.9)	17.6 (6.6–26.5)	190	0.88
sE2 on day of first trigger (pg/mL) (A)	14,876 (6,183–27,998)	10,964 (1,791–33,494)	144	0.15
sE2 on day of second trigger (pg/mL) (B)	10,240 (1,662–28,325)	2,636 (1,104–3,877)	39	<0.001*
E2 rate (B/A) (%)	72.8 (19.6–166.5)	23.7 (11.4–54.1)	44	<0.001*
sP4 on day of first trigger (ng/mL) (C)	3.3 (1.2–8.9)	4.0 (0.6–11.6)	191	0.91
sP4 on day of second trigger (ng/mL) (D)	4.6 (1.7–36.6)	13.0 (3.6–52.2)	118	0.01*
P4 rate (D/C) (%)	105.0 (61.1–1507.0)	396.6 (94.2–642.9)	109	0.009*

**Figure 2:**
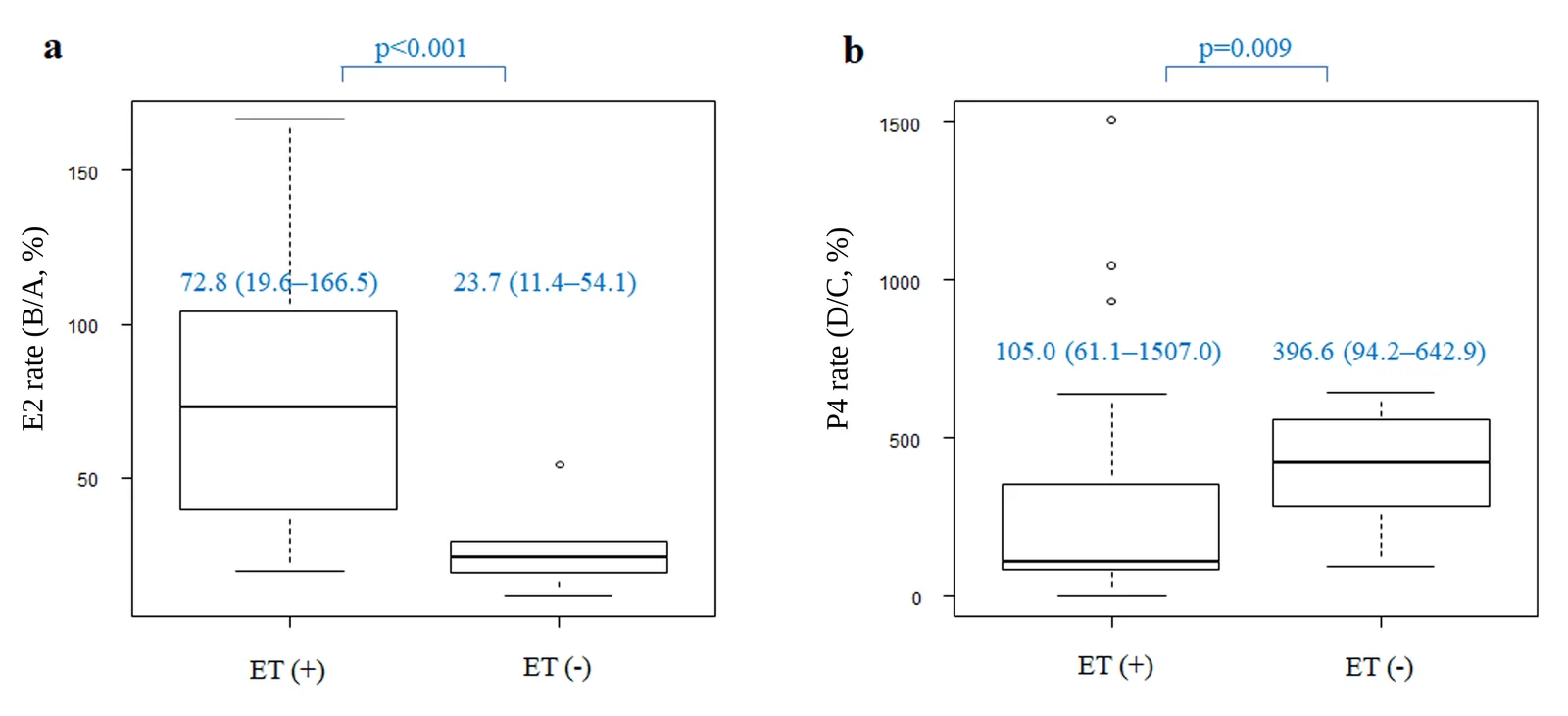
Estradiol and progesterone dynamics between the first and second triggers. (a) E2 rate, defined as the serum estradiol concentration on the day of the second trigger expressed as a percentage of that on the day of the first trigger (B/A in Table 2). Values below 100% indicate a fall in estradiol between the two triggers. (b) P4 rate, defined as the serum progesterone concentration on the day of the second trigger expressed as a percentage of that on the day of the first trigger (D/C in Table 2). Values above 100% indicate a rise in progesterone. Boxes show the median and interquartile range, whiskers extend to the most extreme value within 1.5 times the interquartile range, and circles denote values beyond that. Figures in blue above each box are the median and range. Groups were compared with the Mann-Whitney U test. ET, embryo transfer; E2, estradiol; P4, progesterone.

The E2 rate, defined as the concentration at the second trigger as a proportion of that at the first, was 72.8 (19.6-166.5)% in the ET (+) group and 23.7 (11.4-54.1)% in the ET (−) group (P < 0.001). The corresponding P4 rate was 105.0 (61.1-1507.0)% and 396.6 (94.2-642.9)% (P = 0.009). The relationship between the percentage change in E2 and that in P4 is shown in Figure 2.

Clinical outcomes

The number of oocytes collected following the rescue hCG trigger was 16.5 (4-41) in the ET (+) group and 5.0 (0-22) in the ET (−) group (P < 0.001; Table 3). M2 oocytes numbered 14.0 (3-33) versus 3.0 (0-19) (P = 0.03), while the M2 rate per collected oocyte did not differ. Fertilized oocytes frozen at the pronuclear stage numbered 5.0 (1-21) versus 1.0 (0-7) (P < 0.001), and the fertilization rate per mature oocyte was 50.0 (10-100)% versus 16.7 (0-100)% (P = 0.002). Embryos cultured to the blastocyst stage numbered 3.0 (0-13) versus 1.0 (0-2) (P = 0.002), vitrified blastocysts 1.0 (0-8) versus 0 (P = 0.02), and the blastocyst formation rate 58.3 (0-100)% versus 0% (P = 0.02).

**Table 3:** Clinical outcomes categorized by embryo transfer after the rescue human chorionic gonadotropin protocol. Data are presented as median (range) unless otherwise indicated; OHSS and live birth data are counts (n) or percentages. Continuous variables were compared using the Mann-Whitney U test; the occurrence of moderate OHSS was compared using the chi-squared test with Yates’ continuity correction (df = 1). Statistical significance was defined as p < 0.05, and significant values are marked with an asterisk (*). A hyphen (–) indicates that no statistical comparison was performed for that row. Every patient in the series developed OHSS of some grade; no patient developed severe OHSS. ET, embryo transfer; M2, metaphase II; OHSS, ovarian hyperstimulation syndrome.

	ET (+) (n = 30)	ET (−) (n = 13)	Statistic	p value
No. of retrieved oocytes (A)	16.5 (4–41)	5.0 (0–22)	U = 68	<0.001*
No. of mature (M2) oocytes (B)	14.0 (3–33)	3.0 (0–19)	U = 113	0.03*
M2 rate (B/A) (%)	80.7 (53.8–100)	66.7 (0–100)	U = 153	0.27
No. at the pronuclear stage (C)	5.0 (1–21)	1.0 (0–7)	U = 68	<0.001*
Fertilization rate (C/B) (%)	50.0 (10–100)	16.7 (0–100)	U = 78	0.002*
No. of cultured fertilized eggs (D)	3.0 (0–13)	1.0 (0–2)	U = 78	0.002*
No. of vitrified blastocysts (E)	1.0 (0–8)	0 (0)	U = 107	0.02*
Blastocyst formation rate (E/D) (%)	58.3 (0–100)	0 (0)	U = 107	0.02*
OHSS, mild (n) (F)	19	9	–	–
OHSS, moderate (n) (G)	11	4	–	–
OHSS, severe (n)	0	0	–	–
Moderate OHSS (G/F+G) (%)	36.7	30.8	χ² = 0.001	0.98
Live births (n)	18	0	–	–

Across the whole cohort, oocytes were collected in 37 of 43 cycles (86.0%). Subsequent frozen-thawed embryo transfer under hormone replacement resulted in 18 live births, a live birth rate of 41.9% (18/43) per rescue cycle and 60.0% (18/30) among cycles in which a transferable embryo was obtained. All 18 clinical pregnancies proceeded to live birth.

Within the ET (−) group (n = 13), no oocyte was collected in six cycles. In one cycle, oocytes were collected but none fertilized. In the remaining six, fertilization occurred, but embryo transfer was cancelled because the frozen embryos did not survive thawing or did not reach the morphology required for transfer despite culture from the pronuclear stage.

Onset of OHSS

All 43 patients treated with the rescue hCG protocol developed OHSS. It was mild in 28 (65.1%) and moderate in 15 (34.9%); no patient developed severe OHSS requiring hospitalization. In the ET (+) group, 19 patients had mild and 11 moderate OHSS; in the ET (−) group, nine had mild and four had moderate OHSS. The proportion of moderate OHSS did not differ between the groups (Table 3).

Gonadotropin thresholds

Receiver operating characteristic (ROC) analysis of the dichotomous outcome, taking failure to obtain a transferable embryo as the positive outcome, yielded a baseline LH threshold of 1.9 mIU/mL (AUC 0.83, 95% CI 0.70-0.97) and a baseline FSH threshold of 5.4 mIU/mL (AUC 0.85, 95% CI 0.70-0.99). Applied to the cohort, a baseline LH of 1.9 mIU/mL or above had a sensitivity of 12/13 (0.92) and a specificity of 22/30 (0.73), and a baseline FSH of 5.4 mIU/mL or above had a sensitivity of 12/13 (0.92) and a specificity of 25/30 (0.83). Both thresholds separated the ET (+) and ET (−) groups (P < 0.001 for both; Table 4).

**Table 4:** Contingency tables by outcome and gonadotropin threshold. Values are numbers of cycles. P values were calculated using the chi-squared test with Yates’ continuity correction (df = 1) and were <0.001 for both thresholds. Taking failure to obtain a transferable embryo as the positive outcome, the odds of failure with 95% confidence intervals by Woolf’s method were 33.0 (3.7-296) for a baseline LH of 1.9 mIU/mL or above, and 60.0 (6.3-572) for a baseline FSH of 5.4 mIU/mL or above; the width of these intervals reflects the fact that only one cycle below each threshold failed to yield a transferable embryo. Sensitivity and specificity were 12/13 (0.92) and 22/30 (0.73) for the LH threshold, and 12/13 (0.92) and 25/30 (0.83) for the FSH threshold. ET, embryo transfer; LH, luteinizing hormone; FSH, follicle-stimulating hormone.

Baseline LH	ET (+) (n = 30)	ET (−) (n = 13)	Total
LH < 1.9 mIU/mL	22	1	23
LH ≥ 1.9 mIU/mL	8	12	20
Total	30	13	43
Baseline FSH	ET (+) (n = 30)	ET (−) (n = 13)	Total
FSH < 5.4 mIU/mL	25	1	26
FSH ≥ 5.4 mIU/mL	5	12	17
Total	30	13	43

The odds of failing to obtain a transferable embryo were 33.0 (95% CI 3.7-296) for a baseline LH of 1.9 mIU/mL or above, and 60.0 (95% CI 6.3-572) for a baseline FSH of 5.4 mIU/mL or above. The width of these intervals reflects the fact that only one cycle below each threshold failed to yield a transferable embryo. No multivariable model was fitted, for the reasons given in the Methods.

## Discussion

In this series, a rescue hCG trigger permitted oocyte collection in 37 of 43 cycles and yielded a transferable embryo in 30, in patients whose cycles would otherwise have been cancelled after a failed GnRH agonist trigger. Eighteen live births resulted, a live birth rate of 41.9% per rescue cycle and 60.0% among cycles in which a transferable embryo was obtained. No severe OHSS requiring hospitalization occurred. Baseline LH and FSH, measured before stimulation began, distinguished the cycles that went on to yield a transferable embryo from those that did not, as did the change in serum E2 and P4 between the two triggers.

Empty follicle syndrome (EFS) is a recognized limitation of the GnRH agonist trigger, and the reported incidence varies widely, from 0.045% to 7% [9], reflecting differences in definition as much as in practice. In the present series, the incidence following a GnRH agonist trigger alone was 0.9% (43/4,778 cycles), lower than the 3.3% reported by Deepika et al. [22]; the difference is likely attributable to our restriction of the denominator to cycles triggered with a GnRH agonist alone, and to differences between series in the proportion of patients with polycystic ovarian syndrome. With respect to salvage, Christopoulos et al. [10] reported the first successful rescue hCG trigger with fresh transfer in six patients, Chang et al. [17] re-triggered in 22 cycles, and Deepika et al. [22] achieved oocyte collection in six of nine cases with clinical pregnancy in three. Our salvage rate of 30 transferable embryos in 43 cycles is comparable to that experienced in a considerably larger series. Our practice differs from the earlier reports principally in that all zygotes were cryopreserved at the pronuclear stage, so that no fresh transfer was attempted in a cycle already exposed to two ovulation triggers, and every transfer took place in a subsequent hormone-replacement cycle.

The most useful predictors were available before stimulation began. A baseline LH below 1.9 mIU/mL and a baseline FSH below 5.4 mIU/mL each identified cycles likely to yield a transferable embryo, with only one cycle below each threshold failing to do so. The corresponding odds of failure were 33.0 (95% CI 3.7-296) for a baseline LH at or above 1.9 mIU/mL and 60.0 (95% CI 6.3-572) for a baseline FSH at or above 5.4 mIU/mL. These confidence intervals span two orders of magnitude, and the point estimates should be read as indicating the direction and approximate strength of an association rather than as usable effect sizes.

At first glance, this finding appears to conflict with that of Popovic-Todorovic et al. [23], who identified low serum LH at the start of stimulation as a risk factor for suboptimal oocyte yield following a GnRH agonist trigger. We suggest the two observations are complementary rather than contradictory. A low baseline gonadotropin level predicts failure of the agonist trigger, because the pituitary reserve available for release by the agonist is limited; but the follicles themselves, and their LH and hCG receptors, are unaffected, so an exogenous hCG trigger can act directly and retrieval succeeds. Conversely, when baseline LH and FSH are preserved and the agonist trigger nevertheless fails, the defect is unlikely to be pituitary, and a second trigger acting on the same downstream pathway would not be expected to help. This is consistent with the poor yield observed in our ET (−) group despite adequate baseline gonadotropins.

**Figure 3:**
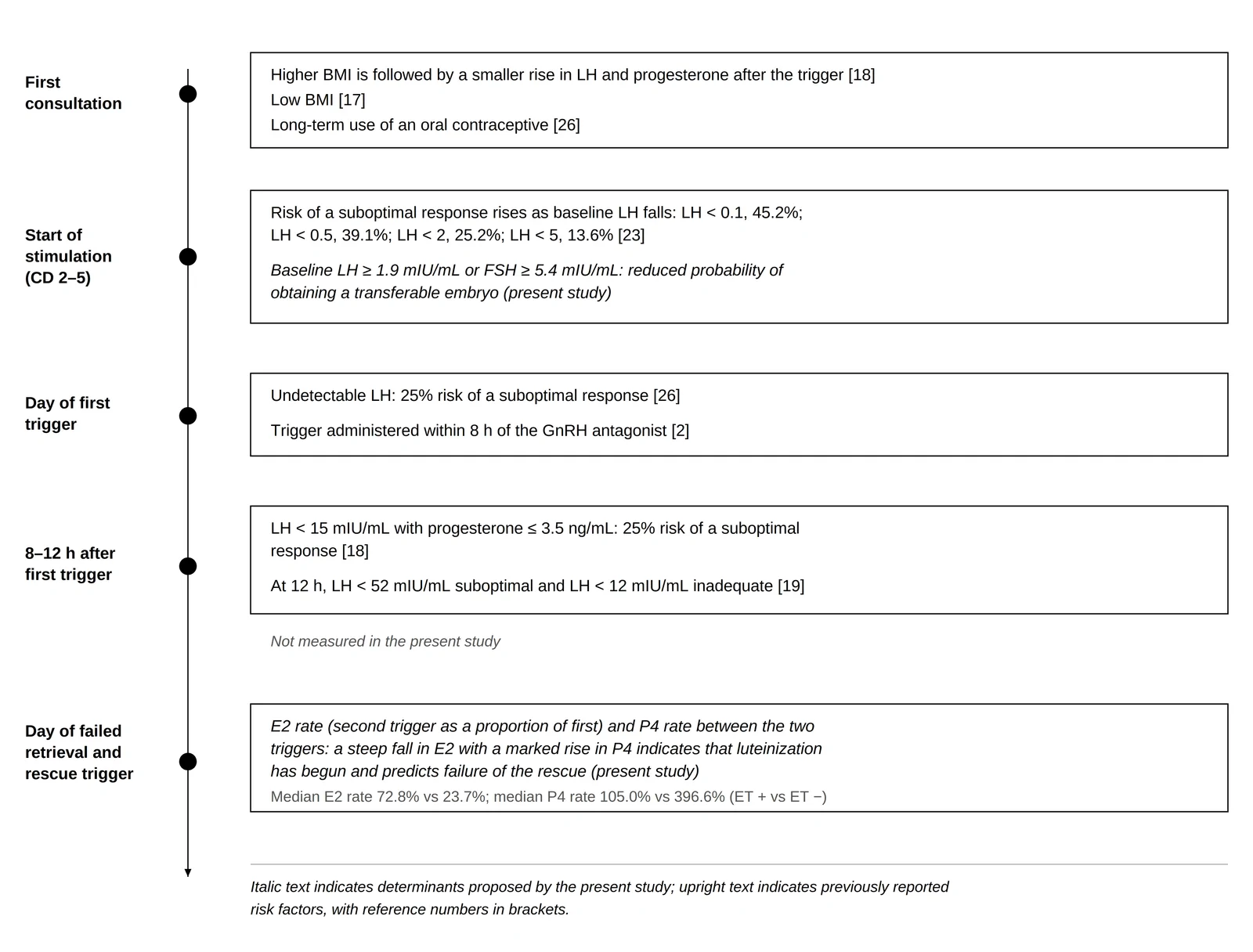
Determinants of the outcome of a rescue human chorionic gonadotropin protocol, by stage of treatment. Each stage of treatment is shown with the determinants of a suboptimal response to the GnRH agonist trigger, or of the outcome of the rescue protocol, that become available at that point. Upright text indicates risk factors reported previously, with reference numbers in brackets; italic text indicates determinants proposed by the present study. The LH response 8-12 h after the first trigger, which discriminates an adequate from an inadequate response in previous work, was not measured in this series. BMI, body mass index; CD, cycle day; LH, luteinizing hormone; FSH, follicle-stimulating hormone; E2, estradiol; P4, progesterone; GnRH, gonadotropin-releasing hormone; ET, embryo transfer.

Determinants of the outcome are distributed across the whole course of treatment rather than concentrated at any single point. Figure 3 sets out those reported previously alongside those proposed here, arranged by the stage of treatment at which each becomes available. Body mass index and previous oral contraceptive use are known before stimulation begins [17,18,23]; baseline gonadotropins are available on cycle days 2-5 [24]; the interval between the antagonist and the trigger, and the LH concentration on the day of the trigger, are determined during stimulation [2,25]; and the LH response 8-12 h after the trigger, which we did not measure, discriminates an adequate from an inadequate response [18,19]. The contribution of the present study falls at the first and the last of these points: baseline LH and FSH before stimulation begins, and the estradiol and progesterone dynamics between the two triggers.

We propose, as a hypothesis for future testing, that failure of a GnRH agonist trigger comprises more than the two categories of the conventional classification into genuine EFS and false EFS. Genuine EFS, in which no oocyte is obtained despite a correctly administered and biologically adequate trigger, is not addressed by our data, since we cannot exclude it in the six ET (−) cycles from which no oocyte was recovered at re-OPU. Within what would conventionally be labelled false EFS, however, our data suggest a gradation rather than a dichotomy.

At one extreme are cycles with low baseline gonadotropin levels, in which the agonist trigger releases insufficient endogenous LH to induce final maturation but the follicles remain fully responsive to exogenous hCG. These cycles showed only a modest change in serum E2 between the two triggers, the E2 concentration at the second trigger being a median of 72.8% of that at the first, and a P4 rise close to unity at a median of 105.0%, consistent with follicles that had not yet begun to luteinize. The oocyte yield after the rescue trigger was good, at a median of 16.5 oocytes, and the outcome favourable. At the other extreme are cycles in which baseline gonadotropins were preserved and the agonist trigger nonetheless produced a partial response. In these, E2 had fallen to a median of 23.7% of the first-trigger value, and P4 had risen to a median of 396.6% by the time the second trigger was given, indicating that luteinization had already begun. The oocyte yield was correspondingly poor, at a median of 5.0, and no transferable embryo was obtained. We interpret this second pattern as a partially effective agonist trigger that initiated maturation without permitting retrieval, leaving follicles that were no longer rescuable by the time hCG was administered. The dynamics of E2 and P4 after trigger have been analyzed previously: P4 increases similarly on the first and second days after either an hCG or a GnRH agonist trigger [24,25], whereas E2 decreases more steeply after a GnRH agonist trigger [24].

The LH values measured on the day of the second trigger are consistent with this interpretation. Because that sample was drawn after the GnRH agonist trigger, it reflects residual LH on the descending limb of the response. The ET (−) group had a higher residual LH than the ET (+) group, a median of 1.3 compared with 0.4 mIU/mL (P = 0.04), and simultaneously the greater P4 rise and steeper E2 fall described above. Read together, these three observations suggest that the cycles that failed were not those in which the agonist trigger did nothing, but those in which it did something insufficient: enough LH was released to initiate luteinization, yet not enough, or not early enough, to permit retrieval of mature oocytes 34-36 h later. The cycles that succeeded with rescue hCG are those in which the pituitary response appears to have been minimal and the follicles were consequently still intact when the second trigger was given.

This proposed gradation is a hypothesis generated by the present data, not a result established by them. It rests on group medians in 30 and 13 cycles,respectively; the intermediate pattern was not pre-specified, and the mechanism we suggest, namely differing hypothalamic and pituitary reactivity to the agonist, was not measured directly. Post-trigger LH kinetics [18,26] would be the appropriate measurement with which to test it, and we did not obtain them.

Several limitations qualify these findings. The most important concerns OHSS. No severe OHSS requiring hospitalization occurred, but with 43 cycles and a background incidence of severe OHSS estimated at 1-5% of stimulated cycles [27], fewer than one case would have been anticipated; the exact upper 95% confidence limit for an observed rate of 0/43 is 8.2%. The absence of severe OHSS in this series is therefore reassuring but cannot be taken as evidence of safety, and a substantially larger series would be required to establish it. A further caution applies. The ET (−) group had a markedly lower E2 concentration at the second trigger, a median of 2,636 compared with 10,240 pg/mL, and a lower oocyte yield, 5.0 compared with 16.5, both of which are established determinants of OHSS risk. Approximately one-third of the cohort was therefore at low risk of severe OHSS for reasons unrelated to the rescue protocol, and the absence of severe cases across the series as a whole cannot be attributed to the protocol without qualification.

It might reasonably be asked how a protocol with no fixed dosing rule can be said to limit OHSS at all. The answer is that in this protocol the safeguards against severe OHSS are structural rather than pharmacological, and they operate independently of the dose chosen for any individual patient. First, the GnRH agonist trigger was used in the first instance precisely because these were cycles at high risk, so that of 4,778 such cycles, 4,735 were completed with no exposure to hCG whatsoever; only the 0.9% in which the agonist trigger failed received any hCG at all. Second, and most importantly, every fertilized oocyte in this series was cryopreserved at the pronuclear stage, and no fresh embryo transfer was performed in the rescue cycle, so that no patient was exposed to the endogenous hCG of an implanting pregnancy. Severe OHSS is predominantly the late-onset form driven by that exposure, and a universal freeze-all policy removes the stimulus that sustains it [1,27]. Third, follicles from one ovary had already been aspirated at the abandoned first retrieval, reducing the granulosa cell mass available to respond to the second trigger. Fourth, the doses actually administered were low: 79.1% of cycles received 3,333 IU or less, which is the observed consequence of a consistent clinical intention to give the least hCG that would work.

It should also be said plainly that OHSS was not avoided in this series. Every one of the 43 patients developed OHSS of some grade: 28 mild and 15 moderate. What did not occur was progression to the severe form requiring hospitalization. The absence of a dosing algorithm is nonetheless a real limitation, and it is a limitation of a specific kind: it means this series cannot be used to recommend a dose, and cannot identify the minimum effective dose of a rescue trigger. That question requires a prospective study with pre-specified dosing, which we would regard as the natural next step.

Second, our first trigger was buserelin acetate administered intranasally by the patient, a route in which both absorption and adherence vary, and no blood was sampled in the 8-12 h window after the trigger in which the LH response is conventionally assessed [18,19]. The LH value we report for the day of the first trigger was drawn before the trigger was given and therefore carries no information about the response to it. We consequently cannot exclude inadequate drug delivery as a contributing cause in some cases, and the series does not distinguish genuine EFS from false EFS attributable to administration failure. Because the two would be expected to differ in their response to a second trigger, this limitation bears directly on our estimate of the salvage rate. The residual LH values discussed above were obtained at a variable interval after the agonist trigger, determined by the timing of the clinic visit rather than by protocol, and they should be regarded as supporting rather than establishing the interpretation we have offered. Prospective series should include a standardized post-trigger LH measurement.

Third, the analysis is exploratory. Thirteen cycles failed to yield a transferable embryo, and only one cycle below each gonadotropin threshold did so; no multivariable model can be supported by such data, and the thresholds were derived from the same cycles in which they were evaluated. They require prospective validation before they are used to select candidates for a rescue protocol. Fourth, because the contralateral ovary was deliberately left unaspirated at the initial retrieval, the series cannot report the oocyte yield that a contralateral aspiration at that procedure would have produced, and therefore cannot quantify what was gained by deferring it. Fifth, all 18 clinical pregnancies proceeded to live birth, with no pregnancy losses; with 18 pregnancies, this observation is compatible with a background loss rate of up to approximately 18% and should not be interpreted as evidence of a reduced risk of miscarriage.

Taken together, these findings suggest that within a GnRH antagonist protocol, a GnRH agonist trigger is followed by successful oocyte retrieval in the great majority of cycles; in our centre, retrieval succeeded in 4,735 of 4,778 such cycles. The failure rate of 0.9% is specific to our protocol and to our operational definition of failure, and rates elsewhere have ranged more widely [9,22]. Failure was concentrated among cycles with low baseline gonadotropin levels. Where re-OPU can be performed with a low hCG dose, a live birth remains achievable, as it was in 18 of the 43 cycles reported here. For patients with a poor outcome after a partial response to the agonist trigger, whether a dual trigger should be selected from the outset is a question our data raise but cannot answer, and it warrants prospective study.

## Conclusions

A rescue hCG trigger permitted oocyte retrieval in 37 of 43 cycles, and a transferable embryo in 30, following failure of a GnRH agonist trigger; 18 live births resulted, a live birth rate of 41.9% per rescue cycle. No severe OHSS occurred, although the size of the series does not permit a conclusion about the risk of severe OHSS, which remains to be established in a larger cohort. In this exploratory analysis, a baseline LH below 1.9 mIU/mL and a baseline FSH below 5.4 mIU/mL identified cycles more likely to yield a transferable embryo; these thresholds were derived from the present data and require prospective validation before clinical use. The differing E2 and P4 dynamics observed between the two triggers suggest that failure of a GnRH agonist trigger may not be a single entity, a hypothesis that post-trigger LH measurement in future series would allow to be tested.
